# Supplementary material for: The interactome of intact mitochondria by cross-linking mass spectrometry provides evidence for coexisting respiratory supercomplexes
Source: Mol Cell Proteomics. 2017 Dec 8;17(2):216–32. doi: 10.1074/mcp.RA117.000470 (PMC5795388; doi:10.1074/mcp.RA117.000470)
Supplement: Supplemental Data [file supp_RA117.000470_133922_0_supp_23978_fzffwf.zip › spectra_annotation/mito_DR_spectra_annotation/216-1-4-1-3-1.pdf]

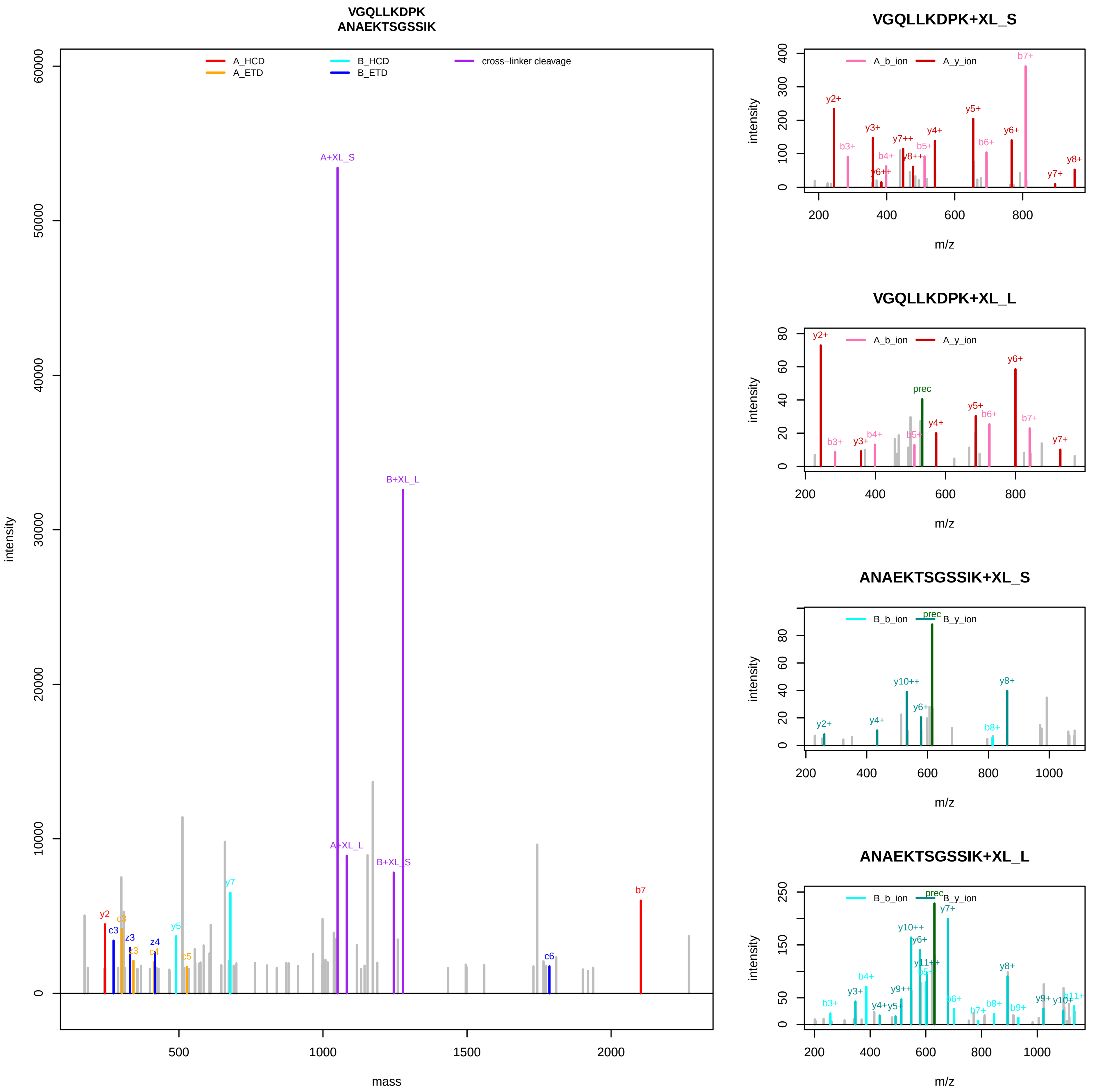
VGQLLKDPK
ANAEKTSGSSIK
A_HCD
A_ETD
B_HCD
B_ETD
cross−linker cleavage
A+XL_S
B+XL_L
A+XL_L
B+XL_S
y2
c3
z3
z4
c4
y5
c5
y7
c6
b7
intensity
mass
VGQLLKDPK+XL_S
A_b_ion
A_y_ion
y2+
b3+
y3+
b4+
y6++
y7++
y8++
b5+
y4+
y5+
b6+
y6+
b7+
y7+
y8+
m/z
VGQLLKDPK+XL_L
prec
ANAEKTSGSSIK+XL_S
B_b_ion
B_y_ion
y10++
y6+
b8+
ANAEKTSGSSIK+XL_L
b9+
y9+
y10+
b11+
y11++
y9++
